# Supplementary material for: Content-rich biological network constructed by mining PubMed abstracts
Source: BMC Bioinformatics. 2004 Oct 8;5:147. doi: 10.1186/1471-2105-5-147 (PMC528731; doi:10.1186/1471-2105-5-147)
Supplement: Additional File 2 — The original results of the above study (non-essential files are deleted to keep the file size under the limit set by BMC bioinformatics). [file 1471-2105-5-147-S2.bz2 › chilibotAdditionalFile2/dip40/457ID10733106E10474/html/CCR3_CCL7.html]

 


 **CCR3** and **CCL7** 
  
Found 58 abstracts in PubMed, retrieved 40.  
 

 What does Google say? 
 PDF only 
| .edu only 

---

**Interactive relationship** (e.g. stimulation, inhibition, etc)

**Neutral relationship**- Therefore, interactions of eotaxin s, eotaxin CCL11, RANTES CCL5, and MCP 1 CCL2, MCP 2 CCL8, MCP 3  [ **CCL7** ]   **CCL7** , MCP 4 CCL13 with  **CCR3**  are responsible for the recruitment of basophils, eosinophils and mast cells.  Ref: 12009564 Mol Immunol, 2002
- Comparison of the structure of vMIP II with eotaxin 1, RANTES, and MCP 3  [ **CCL7** ]  suggests a unique mechanism for  **CCR3**  activation.  Ref: 11041848 Biochemistry, 2000
- Surface epitopes identified on RANTES to be involved in binding to  **CCR3**  are mimicked on the eotaxin 1 and MCP 3  [ **CCL7** ]  surface.  Ref: 11041848 Biochemistry, 2000
- Ligands for CCR2 and  **CCR3**  include MCP 1 and MCP 3  [ **CCL7** ]  which were co localised around vessels with the infiltrating leukocytes, but were also present in unaffected areas of cortex.  Ref: 10900353 J Neuroimmunol, 2000
- The activation of the target cells by MCP 3  [ **CCL7** ]  is via specific chemokine receptors CCR2 and  **CCR3** , of which CCR2 is shared with MCP 1.  Ref: 10564716 Biochim Biophys Acta, 2000
- The results presented here indicate that the structural basis for interaction with the chemokine receptor  **CCR3**  by vMIP II is different from that for the physiological agonists eotaxin 1, RANTES, and MCP 3  [ **CCL7** ] .  Ref: 11041848 Biochemistry, 2000
- **CCR3**  is known to be a ligand for CC chemokines such as RANTES, eotaxin and monocyte chemotactic protein 3  [ **CCL7** ]  MCP 3  [ **CCL7** ] .  Ref: 11240271 J Dermatol Sci, 2001
- **CCR3**  ligands, i.e., regulated on activation normal T cell expressed and secreted RANTES, eotaxin, eotaxin 2, and monocyte chemoattractant protein MCP 3  [ **CCL7** ] , induced significant migration, while other chemokines showed no significant migration inducing ability.  Ref: 10505457 Allergy, 1999
- I TAC, Mig, and IP10 compete for the binding of eotaxin to  **CCR3**  bearing cells and inhibit migration and Ca 2 changes induced in such cells by stimulation with eotaxin, eotaxin 2, MCP 2 monocyte chemottractant protein 2, MCP 3  [ **CCL7** ] , MCP 4, and RANTES regulated on activation normal T cell expressed and secreted.  Ref: 11110785 J Biol Chem, 2001

**Non-interactive relationship** (e.g. studied together, co-existance, homology, etc.)

- The chemokine receptor  **CCR3**  and ligands monocyte chemoattractant protein MCP 3  [ **CCL7** ] , MCP 4, regulated upon activation normal T cell expressed and secreted RANTES and eotaxins I and II are of particular relevance for the recruitment and activation of eosinophils.  Ref: 11340325 Int Arch Allergy Immunol, 2001
- Eosinophil chemotactic chemokines eotaxin, eotaxin 2, RANTES, monocyte chemoattractant protein 3  [ **CCL7** ]  MCP 3  [ **CCL7** ] , and MCP 4, and C C chemokine receptor 3  [ **CCR3** ]  expression in bronchial biopsies from atopic and nonatopic Intrinsic asthmatics.  Ref: 10570327 J Immunol, 1999
- Our findings point to a role for C C chemokine receptor 2 CCR2 and  **CCR3**  agonists such as monocyte chemotactic proteins MCPs 1 CCL  **CCL7**  and 5 CCL12 as important participants that are subject to regulation by Th2 cytokines interleukin IL 4 and IL 13.  Ref: 12102713 Parasite Immunol, 2002
- O 3 increased lung protein levels of CXCL10,  **CCL7** , and  **CCR3**  CCL7R.  Ref: 11777981 J Immunol, 2002
- However, degranulation was induced only by  **CCR3**  ligands, including MCP 3  [ **CCL7** ] , MCP 4, RANTES, and eotaxin.  Ref: 10984371 J Allergy Clin Immunol, 2000
- OBJECTIVE AND DESIGN The gene expression profile of  **CCR3**  ligands, eotaxin, RANTES, and monocyte chemoattractant protein 3  [ **CCL7** ]  MCP 3  [ **CCL7** ] , was examined in normal and inflamed guinea pig lungs.  Ref: 11822789 Inflamm Res, 2001
- We investigated tissue samples and tumor cell suspensions of patients with CD30 CTCL and CD30 CTCL for expression of the chemokine receptors  **CCR3** , CCR4, and CCR8 and the  **CCR3**  ligands eotaxin CCL11, monocyte chemoattractant protein 3  [ **CCL7** ]   **CCL7** , and RANTES CCL5.  Ref: 12393570 Blood, 2003
- Other human  **CCR3**  agonists, such as MCP 3  [ **CCL7** ] , RANTES and MCP 4 failed to increase dermal eosinophil peroxidase activity at doses up to 1 microg site 1 whereas the latter did produce a small effect at 3 microg site 1.  Ref: 10516663 Br J Pharmacol, 1999
- These ligands include the  **CCR3**  selective chemokines eotaxin and eotaxin 2 and the more promiscuous CC chemokines, MCP 4, MCP 3  [ **CCL7** ] , MCP 2 and RANTES.  Ref: 11306952 Int Arch Allergy Immunol< MedlineTA>Int Arch Allergy Immunol, 1999
- We have compared the surface topology and electrostatic potential of vMIP II to those of eotaxin 1, RANTES, and MCP 3  [ **CCL7** ] , three  **CCR3**  physiological agonists with known three dimensional structures.  Ref: 11041848 Biochemistry, 2000
- Compared with AC and NC, the numbers of EG2 cells and the cells expressing mRNA for eotaxin, eotaxin 2, RANTES, MCP 3  [ **CCL7** ] , MCP 4, and  **CCR3**  were significantly increased in AA and NAA p <.  Ref: 10570327 J Immunol, 1999
- We studied the expression of eotaxin and  **CCR3** , as well as MCP 3  [ **CCL7** ] , MIP 1alpha and interleukin 8, in atopic dermatitis and normal skin by immunohistochemistry and nested reverse transcriptase polymerase chain reaction.  Ref: 10417617 J Invest Dermatol, 1999
- Immunohistochemistry for MIP 1beta, MIP 1alpha, RANTES, eotaxin, and MCP 3  [ **CCL7** ]  ligands for CCR5 and or  **CCR3**  revealed the presence of MIP 1beta predominantly in a subpopulation of reactive astrocytes, which were more widespread in AD than control brains, and MIP 1alpha predominantly in neurons and weakly in some microglia in both AD and controls.  Ref: 9665462 Am J Pathol, 1998
- Several CC chemokines including eotaxin CCL 11, eotaxin 2 CCL 24, RANTES CCL 5, and monocyte chemotactic protein 3  [ **CCL7** ]  MCP 3  [ **CCL7** ] , CCL 7  [ **CCL7** ]  and 4 MCP 4, CCL 13 are potent eosinophil chemotactic and activating peptides acting through CC chemokine receptor 3  [ **CCR3** ]   **CCR3** .  Ref: 10969084 J Biol Chem, 2000
- The beta chemokine receptor  **CCR3** , which recognizes the ligands eotaxin, eotaxin 2, monocyte chemotactic protein 3  [ **CCL7** ] , MCP4, and RANTES, plays a central role in this process, and antagonists to this receptor could have potential therapeutic use in the treatment of allergy.  Ref: 10640766 J Immunol, 2000
- In contrast, responses to MCP 3  [ **CCL7** ]  were blocked only in the presence of both mAbs, but not after pretreatment with either anti CCR2 or anti  **CCR3**  mAb alone.  Ref: 11120855 J Immunol, 2000
- We examined the expression of eotaxin, eotaxin 2, RANTES, monocyte chemoattractant protein 3  [ **CCL7** ]  MCP 3  [ **CCL7** ] , MCP 4, and  **CCR3**  in the bronchial mucosa from atopic AA and nonatopic intrinsic.  Ref: 10570327 J Immunol, 1999
